# Kernel-based geographically and temporally weighted autoregressive model for house price estimation

**DOI:** 10.1371/journal.pone.0205063

**Published:** 2018-10-11

**Authors:** Jooyong Shim, Changha Hwang

**Affiliations:** 1 Department of Statistics, Inje University, Gimhae, Korea; 2 Department of Applied Statistics, Dankook University, Yongin, Korea; Central South University, CHINA

## Abstract

Spatiotemporal nonstationarity and autocorrelation are two crucial points in modeling geographical data. Previous studies have demonstrated that geographically and temporally weighted autoregressive (GTWAR) model accounts for both spatiotemporal nonstationarity and autocorrelation simultaneously to estimate house prices. Therefore, this paper proposes a kernel-based GTWAR (KBGTWAR) model by incorporating the basic principle of support vector machine regression into spatially and temporally varying coefficients model. The efficacy of KBGTWAR model is demonstrated through a case study on housing prices in the city of Shenzhen, China, from year 2004 to 2008. Comparing the existing models, KBGTWAR model obtains the lowest value for the residual sum of squares (RSS) and the highest value for the coefficient of determination *R*^2^. Moreover, KBGTWAR model improves the goodness of fit of the existing GTWAR model from 12.0 to 4.5 in terms of RSS, from 0.914 to 0.968 in terms of *R*^2^ and from 3.84 to 4.45 in terms of F-statistic. The results show that KBGTWAR model provides a comparatively high goodness of fit and sufficient explanatory power for both spatiotemporal nonstationarity and autocorrelation. The results of this study demonstrate that the proposed KBGTWAR model can be used to effectively formulate polices for real estate management.

## Introduction

Analysis of relationships between an output variable and input variables in spatiotemporal fields has recently attracted attention in a data analysis community. Spatiotemporal models occur when data are obtained across time as well as space. Spatial or temporal autocorrelation exists between the observations. In addition, spatial or temporal nonstationarity arises in the relationships. Ordinary regression models have neglected these issues that violate the standard Gauss-Markov assumptions. Spatial econometrics models have been proposed to deal with these issues. Spatiotemporal regression models can contribute to better understanding complex phenomena studied in geographical information science and other fields.

Spatial models not only can account for spatial autocorrelation but also can deal with spatial lag dependence or/and spatial error dependence. There are two approaches for handling spatial autocorrelation. The first is the weight matrix approach, which uses a spatial weight matrix to deal with the spatial relationships between observations. This approach is based on the work of [1]. See for details [2] and [3]. The second is the geostatistical approach, which directly models the covariance matrix of the error terms. This approach is based on the work of [4]. See for details [5] and [6]. Moreover, [3] proposed a spatiotemporal autoregressive (STAR) model to incorporate temporal correlations between observations and found it powerful in the context of residential real estate. [7] developed a two-order STAR model with a Bayesian procedure to effectively detect and correct heteroscedasticity among the residuals.

The spatial models aforementioned have made remarkable contributions to effectively applying spatial and temporal process to regression modeling. However, these models assumed that a global level relationship exists across the study area. By the way, the assumption of stationary or structural stability over space and time is often impractical, because parameters tend to change depending on the study area. To deal with spatial heterogeneity in housing markets, a variety of localized modeling techniques have been proposed. As a result, [8] and [9] demonstrated the usefulness of locally weighted regression in modeling nonmonocentric cities. [10], [11] and [12] proposed geographically weighted regression (GWR), which has grown in popularity amongst real estate modeling methods. GWR model is a local spatial model for exploring spatial nonstationarity. To capture both spatial nonstationarity and spatial autocorrelation in a complex process, [13] proposed a GWR with spatially lagged variables to utilize the spatial variations. But this model did not consider temporal information. To model the effects of temporal heterogeneity, [14] and [15] proposed a geographically and temporally weighted regression (GTWR) to simultaneously capture both spatial and temporal nonstationarity by integrating temporal effects into the traditional GWR model. [16] developed a GTWR model based on travel time distance metrics. But these GTWR models do not consider spatial autocorrelation.

[17] asserted that it is better to deal with both spatial and temporal heterogeneity along with spatial autocorrelation effects in a mixed model. It is because spatial heterogeneity and autocorrelation are generally related in the context of modeling although the two problems are theoretically distinguished. However, it is not easy to build an integrated model with spatiotemporal autocorrelated and heterogeneous effects. [18] proposed a geographically and temporally weighted autoregressive (GTWAR) model to deal with spatiotemporal variations. In this paper we propose an efficient kernel-based GTWAR (KBGTWAR) model by incorporating the basic principle of support vector machine (SVM) regression into spatially and temporally varying coefficients model. SVM, first developed by [19] and his group at AT&T Bell Laboratories, has been successfully applied to a number of real world problems related to classification and regression problems.

The rest of this paper is organized as follows. Section 2 briefly describes the basic principle of GTWR and GTWAR models. Section 3 proposes an efficient KBGTWAR model. Section 4 and section 5 present case study and conclusion, respectively.

## GTWAR model

In this section we briefly review the basic framework for GTWR and GTWAR models. We also review how to determine the adjustment parameters related with these two models.

### GTWR model

The GWR model is a spatially varying coefficient regression approach for exploring spatial nonstationarity of a regression relationship for spatial data [10, 11, 12]. The GWR model extends the traditional linear regression model by allowing local rather than global parameters to be estimated. [14] developed a GTWR model to deal with spatial and temporal nonstationarity simultaneously by integrating temporal effects into the GWR model. [15] developed a GTWR model focusing on spatiotemporal kernel function definition and spatiotemporal bandwidth optimization. [16] developed a GTWR model based on non-Euclidean travel distance. The GTWR model can be expressed as follows:

$$\begin{array}{c} y_{i}=\beta_{0}\left(u_{i},v_{i},t_{i}\right)+\sum_{k=1}^{d}\beta_{k}\left(u_{i},v_{i},t_{i}\right)x_{ki}+\epsilon_{i},i=1,\cdots ,n, \end{array}$$ (1)

where (*u*_*i*_, *v*_*i*_, *t*_*i*_) is the coordinate of the observation *i* in space (*u*_*i*_, *v*_*i*_) at time *t*_*i*_, *β*_0_(*u*_*i*_, *v*_*i*_, *t*_*i*_) indicates the intercept value, *β*_*k*_(*u*_*i*_, *v*_*i*_, *t*_*i*_) indicates the slope for each variable *k* and each space-time point *i*, and *ϵ*_*i*_ represents the random error with no correlation between different points. Here, local parameters *β*_0_(*u*_*i*_, *v*_*i*_, *t*_*i*_) and *β*_*k*_(*u*_*i*_, *v*_*i*_, *t*_*i*_) are continuous functions of the point (*u*_*i*_, *v*_*i*_, *t*_*i*_). We notice that the GTWR model (1) is a spatially and temporally varying coefficient model.

For a given data set, these local parameters are estimated using the weighted least square procedure. The relevant weights *W*_*ij*_, for *j* = 1, ⋯, *n*, indicate the proximity of each data point to the point (*u*_*i*_, *v*_*i*_, *t*_*i*_). Let

$$\begin{array}{c} \beta \left(u_{i},v_{i},t_{i}\right)={\left(\beta_{0}\left(u_{1},v_{1},t_{1}\right),\beta_{1}\left(u_{1},v_{1},t_{1}\right),\cdots ,\beta_{d}\left(u_{1},v_{1},t_{1}\right)\right)}^{t} \end{array}$$ (2)

be the vector of the local parameters for the space-time point *i*. Here, the superscript *t* represents the transpose of vector or matrix. Then, ***β***(*u_i_*, *v_i_*, *t_i_*) is estimated by

$$\begin{array}{c} \hat{\beta }\left(u_{i},v_{i},t_{i}\right)={\left(X^{t}W\left(u_{i},v_{i},t_{i}\right)X\right)}^{-1}X^{t}W\left(u_{i},v_{i},t_{i}\right)y, \end{array}$$ (3)

where ***X*** is the *n* × (*d* + 1) matrix of input variables, ***y*** is the *n*-dimensional vector of output variable, and ***W***(*u_i_*, *v_i_*, *t_i_*) is an *n* × *n* weighting matrix of the form

$$\begin{array}{c} W\left(u_{i},v_{i},t_{i}\right)=\text{diag}\{W_{i1},W_{i2},\cdots ,W_{in}\}. \end{array}$$ (4)

In addition, the fitted value $\hat{y}$ is obtained as follows:

$$\begin{array}{c} \hat{y}=(\begin{array}{c} {\hat{y}}_{1}\\ {\hat{y}}_{2}\\ \vdots \\ {\hat{y}}_{n} \end{array})=(\begin{array}{c} x_{1}^{a}{\left(X^{t}W\left(u_{1},v_{1},t_{1}\right)X\right)}^{-1}X^{t}W\left(u_{1},v_{1},t_{1}\right)\\ x_{2}^{a}{\left(X^{t}W\left(u_{2},v_{2},t_{2}\right)X\right)}^{-1}X^{t}W\left(u_{2},v_{2},t_{2}\right)\\ \vdots \\ x_{n}^{a}{\left(X^{t}W\left(u_{n},v_{n},t_{n}\right)X\right)}^{-1}X^{t}W\left(u_{n},v_{n},t_{n}\right) \end{array})y, \end{array}$$ (5)

where $x_{i}^{a}$ is the *i*th row of the matrix ***X*** such that $x_{i}^{a}=\left(1,x_{1i},\cdots ,x_{di}\right)$.

The weights *W*_*ij*_ are usually obtained through an adaptive kernel function. The adaptive kernel function attempts to adjust for the density of data points. This adaptive kernel function could use the same number of observed points in each local neighborhood set. The most commonly used adaptive kernel function is the Gaussian function

$$\begin{array}{c} W_{ij}=\text{exp}(-\frac{d_{ij}^{2}}{h^{2}}), \end{array}$$ (6)

where *d*_*ij*_ is a spatiotemporal distance between points *i* and *j* and *h* is a nonnegative parameter known as bandwidth, which produces a decay of influence with distance. When the spatial and temporal distances between points *i* and *j* are given by ${\left(d_{ij}^{S}\right)}^{2}={\left(u_{i}-u_{j}\right)}^{2}+{\left(v_{i}-v_{j}\right)}^{2},{\left(d_{ij}^{T}\right)}^{2}={\left(t_{i}-t_{j}\right)}^{2}$, we can construct the spatiotemporal distance as as a linear combination between ${\left(d_{ij}^{S}\right)}^{2}$ and ${\left(d_{ij}^{T}\right)}^{2}$ as follows:

$$\begin{array}{c} {\left(d_{ij}^{ST}\right)}^{2}=\mu^{S}[{\left(u_{i}-u_{j}\right)}^{2}+{\left(v_{i}-v_{j}\right)}^{2}]+\mu^{T}{\left(t_{i}-t_{j}\right)}^{2}, \end{array}$$ (7)

where *μ*^*S*^ represents the scale factor of spatial distance and *μ*^*T*^ represents the scale factor of temporal distance. Thus, the weights *W*_*ij*_ can be expressed as

$$\begin{array}{cc} W_{ij} & =\text{exp}\{-(\frac{\mu^{S}[{\left(u_{i}-u_{j}\right)}^{2}+{\left(v_{i}-v_{j}\right)}^{2}]+\mu^{T}{\left(t_{i}-t_{j}\right)}^{2}}{h_{ST}^{2}})\}\\ & =\text{exp}\{-(\frac{{\left(u_{i}-u_{j}\right)}^{2}+{\left(v_{i}-v_{j}\right)}^{2}}{h_{S}^{2}}+\frac{{\left(t_{i}-t_{j}\right)}^{2}}{h_{T}^{2}})\}\\ & =\text{exp}\{-(\frac{{\left(d_{ij}^{S}\right)}^{2}}{h_{S}^{2}}+\frac{{\left(d_{ij}^{T}\right)}^{2}}{h_{T}^{2}})\} \end{array}$$ (8)

$$\begin{array}{ccc} & = & W_{ij}^{S}\times W_{ij}^{T}, \end{array}$$ (9)

where $h_{ST}^{2},h_{S}^{2}=h_{ST}^{2}/\mu^{S}$ and $h_{T}^{2}=h_{ST}^{2}/\mu^{T}$ are the parameters of spatiotemporal, spatial, and temporal bandwidths, respectively. Thus, if the spatial and temporal bandwidths are determined, the weight matrix $W(u_{i},v_{i},t_{i})$ and $\hat{\beta }\left(u_{i},v_{i},t_{i}\right)$ can be obtained.

[18] developed the improved GTWR (IGTWR) with the following spatiotemporal distance

$$\begin{array}{c} \left\{ \begin{array}{cc} d_{ij}^{ST}=\mu^{S}d_{ij}^{S}+\mu^{T}d_{ij}^{T}+2\sqrt{\mu^{S}\mu^{T}d_{ij}^{S}d_{ij}^{T}}\cos\left(\nu \right), & t_{j}<t_{i}\\ d_{ij}^{ST}=\infty , & t_{j}>t_{i} \end{array},\right. \end{array}$$ (10)

where *ν* ∈ [0, *π*]. The adjustment parameters *μ*^*S*^, *μ*^*T*^ and *ν* should be determined.

### GTWAR model

To account for both spatiotemporal heterogeneity and spatial autocorrelation effects simultaneously, [18] developed a GTWAR, which combines the IGTWR model with the autocorrelation regression model. Spatial autocorrelation is considered by introducing the spatial lag $\sum_{j=1}^{n}{\bar{W}}_{ij}y_{j}$ in a linear regression relationship [20].

$$\begin{array}{c} y_{i}=\rho_{i}\sum_{j=1}^{n}{\bar{W}}_{ij}y_{j}+\epsilon_{i},i=1,2,\cdots ,n, \end{array}$$ (11)

where *ρ*_*i*_ represents a spatial autoregressive parameter varying across geographical locations, *ϵ*_*i*_ denotes the random error in the relationship, and ${\bar{W}}_{ij}$ is the element in the *i*th row and *j*th column of the *n* × *n* spatial weight matrix $\bar{W}$ with ${\bar{W}}_{ii}=0$. The elements ${\bar{W}}_{ij}$ are typically row-normalized, such that for each *i*, $\sum_{j=1}^{n}{\bar{W}}_{ij}=1$. Consequently, the spatial lag may be interpreted as a weighted average of the neighbors. We notice that the model (11) is a locally based autoregressive model. Incorporating (11) into the IGTWR model, [18] proposed the following GTWAR model:

$$\begin{array}{c} y_{i}=\beta_{0}\left(u_{i},v_{i},t_{i}\right)+\rho \left(u_{i},v_{i},t_{i}\right)\sum_{j=1}^{n}{\bar{W}}_{ij}y_{j}+\sum_{k=1}^{d}\beta_{k}\left(u_{i},v_{i},t_{i}\right)x_{ki}+\epsilon_{i},i=1,\cdots ,n, \end{array}$$ (12)

where *ρ*(*u*_*i*_, *v*_*i*_, *t*_*i*_) is a scalar spatiotemporal autoregressive parameter at point *i*.

### Model selection

Parameter estimation in GTWR and GTWAR is highly dependent on the adjustment parameters *μ*^*S*^, *μ*^*T*^ and/or *ν* associated with the weighting function used. The selection of the adjustment parameters for GTWR and GTWAR can be determined using a cross validation (CV) approach or the corrected Akaike information criterion (AIC) from [12]. Since the adaptive kernel function generally uses the same number *q* of the nearest observed points, there is one more adjustment parameter. To obtain an optimal value of *q*, the CV or corrected AIC approach could be used. [14] argue that only the parameter ratio *τ* = *μ*^*T*^/*μ*^*S*^ plays an important role in constructing weights. Hence, they set *μ*^*S*^ = 1 to reduce the number of adjustment parameters, and so only three parameters, *q*, *μ*^*T*^ and *ν*.

The CV function is defined as

$$\begin{array}{c} CV\left(\lambda \right)=\sum_{i=1}^{n}{\left(y_{i}-{\hat{y}}^{\left(-i\right)}\left(\lambda \right)\right)}^{2}, \end{array}$$ (13)

where **λ** is the vector of adjustment parameters associated with the GTWR or GTWAR model and ${\hat{y}}^{\left(-i\right)}\left(\lambda \right)$ is the fitted value of *y*_*i*_ with the observation *i* omitted from the calibration process. The corrected AIC function is defined according to [21] as follows:

$$\begin{array}{c} AIC_{c}\left(\lambda \right)=n\log(\frac{RSS(\lambda )}{n})+n\log\left(2\pi \right)+n(\frac{n+\text{tr}(H(\lambda ))}{n-2-\text{tr}(H(\lambda ))}), \end{array}$$ (14)

where *n* is the number of data points in data set, *RSS* is the residual sum of squares defined as $RSS\left(\lambda \right)=\sum_{i=1}^{n}{\left(y_{i}-{\hat{y}}_{i}\right)}^{2}$, and tr(***H***(**λ**)) is the trace of the hat matrix ***H***(**λ**) associated with the GTWR or GTWAR model with the adjustment parameter vector **λ**, which satisfies the relationship $\hat{y}=H\left(\lambda \right)y$. See (5) for the hat matrix related with the GTWR model and [18] for the hat matrix related with the GTWAR model. Here log denotes the natural logarithm. The adjustment parameters are achieved automatically with an optimization technique by minimizing the CV function (13) or the corrected AIC function (14).

## KBGTWAR model

In this section we review SVM regression and illustrate how to develop KBGTWAR model. The underlying idea of KBGTWAR model is that the true mean specification is approximated by combining linear SVM regression with nonlinear feature mapping function of the coordinate vector (*u*_*i*_, *v*_*i*_, *t*_*i*_) of the observed point *i*.

### SVM regression

The foundations of SVM have been originally proposed by [19] and are gaining popularity due to many attractive features, and promising empirical performance. We now briefly review SVM regression. See for details [22]. Suppose we are given the data set $D={\left\{\left(x_{i},y_{i}\right)\right\}}_{i=1}^{n}$ with each covariate vector $x_{i}\in R^{d}$ and the output $y_{i}\in R$. We basically illustrate the case of the linear SVM regression, taking the form

$$\begin{array}{c} f\left(x\right)=w^{t}x+b, \end{array}$$ (15)

where *b* is the bias term. The goal of the linear SVM regression is to find a linear regression function f(x) that approximates all pairs $\left(x_{i},y_{i}\right)$ with *ϵ* precision and is simultaneously as flat as possible. Flatness means that we seek a small ***w***. One way to guarantee this is to minimize the norm ${∥w∥}^{2}$. To make it feasible, we introduce slack variables $\xi_{i},\xi_{i}^{*}$ representing upper and lower constraints on the outputs. This leads to the convex optimization problem

$$\begin{array}{ccc} & & \min_{w,b,\xi ,\xi^{*}}J=\frac{1}{2}{∥w∥}^{2}+C\sum_{i=1}^{n}(\xi_{i}+\xi_{i}^{*})\\ & & \text{subject}\;\text{to}\;\;\{\begin{array}{c} y_{i}-w^{t}x_{i}-b\leq \epsilon +\xi_{i}\\ w^{t}x_{i}+b-y_{i}\leq \epsilon +\xi_{i}^{*}\\ \xi_{i},\xi_{i}^{*}\geq 0 \end{array}. \end{array}$$ (16)

The regularization parameter *C* > 0 determines the trade-off between the flatness of *f* and the fitting errors.

The key idea is to construct the primal Lagrange function

$$\begin{array}{ccc} L=J & - & \sum_{i=1}^{n}\alpha_{i}(\epsilon +\xi_{i}-y_{i}+w^{t}x_{i}+b)\\ & - & \sum_{i=1}^{n}\alpha_{i}^{*}(\epsilon +\xi_{i}^{*}+y_{i}-w^{t}x_{i}-b)-\sum_{i=1}^{n}\left(\eta_{i}\xi_{i}+\eta_{i}^{*}\xi_{i}^{*}\right), \end{array}$$ (17)

where $\alpha_{i},\alpha_{i}^{*},\eta_{i},\eta_{i}^{*}\geq 0$ are Lagrange multipliers. The conditions for optimality are given by

$$\begin{array}{ccc} \frac{\partial L}{\partial w}=0 & \to & w=\sum_{i=1}^{n}\left(\alpha_{i}-\alpha_{i}^{*}\right)x_{i} \end{array}$$ (18)

$$\begin{array}{ccc} \frac{\partial L}{\partial b}=0 & \to & \sum_{i=1}^{n}\left(\alpha_{i}-\alpha_{i}^{*}\right)=0 \end{array}$$ (19)

$$\begin{array}{ccc} \frac{\partial L}{\partial \xi_{i}^{\left(*\right)}}=0 & \to & C-\alpha_{i}^{\left(*\right)}-\eta_{i}^{\left(*\right)}=0,\;i=1,\cdots ,n \end{array}$$ (20)

Here, we refer to *α*_*i*_ and $\alpha_{i}^{*}$ by $\alpha_{i}^{\left(*\right)}$.

Substituting (18), (19) and (20) into (17) yields the dual optimization problem.

$$\begin{array}{ccc} & & \min_{\alpha_{i},\alpha_{i}^{*}}\frac{1}{2}\sum_{i,j=1}^{n}\left(\alpha_{i}-\alpha_{i}^{*}\right)\left(\alpha_{j}-\alpha_{j}^{*}\right)x_{i}^{t}x_{j}+\epsilon \sum_{i=1}^{n}\left(\alpha_{i}+\alpha_{i}^{*}\right)-\sum_{i=1}^{n}y_{i}\left(\alpha_{i}-\alpha_{i}^{*}\right)\\ & & \text{subject}\;\text{to}\;\;\sum_{i=1}^{n}\left(\alpha_{i}-\alpha_{i}^{*}\right)=0\;\;\text{and}\;\;\alpha_{i},\alpha_{i}^{*}\in \left[0,C\right] \end{array}$$ (21)

Solving the above optimization problem determines the Lagrange multipliers ${\hat{\alpha }}_{i},{\hat{\alpha }}_{i}^{*}$. Thus the optimal regression function can be rewritten as follows:

$$\begin{array}{c} \hat{f}\left(x\right)=\sum_{i=1}^{n}\left({\hat{\alpha }}_{i}-{\hat{\alpha }}_{i}^{*}\right)x_{i}^{t}x+\hat{b}. \end{array}$$ (22)

Here, the optimal value of *b* is obtained by employing Karush-Kuhn-Tucker [23] conditions as follows:

$$\begin{array}{c} \hat{b}=\frac{1}{n_{s}}\sum_{i\in I_{\text{sv}}}(y_{i}-\sum_{j=1}^{n}\left({\hat{\alpha }}_{j}-{\hat{\alpha }}_{j}^{*}\right)x_{j}^{t}x_{i}-\epsilon \times \text{sign}\left({\hat{\alpha }}_{i}-{\hat{\alpha }}_{i}^{*}\right)), \end{array}$$ (23)

where *n*_*s*_ is the size of $I_{\text{sv}}=\{i=1,2,\cdots ,n:0<|\alpha_{i}-\alpha_{i}^{*}\left|<C\right\}$.

We now consider how to make the linear SVM regression algorithm nonlinear. This could be achieved by simply preprocessing the the input vectors ***x***_*i*_ by a nonlinear feature mapping function $\phi :R^{d}\to F$ into some feature space F, and then applying the linear SVM regression algorithm. Thus, we only need to use the kernel trick *K*(***x***_*i*_, ***x***_*j*_) = *ϕ*(***x***_*i*_)^*t*^ *ϕ*(***x***_*j*_) in the Eqs (21), (22) and (23) associated with the linear SVM regression algorithm [24]. We never need to know explicitly what *ϕ* is. The most popular kernel is Gaussian kernel defined by

$$\begin{array}{c} K\left(x_{i},x_{j}\right)=\text{exp}(-∥x_{i}-x_{j}{∥}^{2}/2\kappa ),\;i,j=1,\cdots ,n, \end{array}$$ (24)

where *κ* > 0 is the kernel parameter.

### KBGTWAR model

Given the data set $D={\left\{\left(u_{i},x_{i},y_{i}\right)\right\}}_{i=1}^{n}$ with each coordinate vector ***u***_*i*_ = (*u_i_*, *v_i_*, *t_i_*), covariate vector $x_{i}\in R^{d}$ and the output $y_{i}\in R$, we consider the following GTWAR model reexpressed from (12):

$$\begin{array}{c} y_{i}=\rho \left(u_{i}\right)\sum_{j=1}^{n}{\bar{W}}_{ij}y_{j}+\sum_{k=0}^{d}\beta_{k}\left(u_{i}\right)x_{ki}+\epsilon_{i},i=1,\cdots ,n, \end{array}$$ (25)

where *x*_0*i*_ = 1 and *x*_*ki*_ is the *k*th component of ***x***_*i*_ = (*x*_1*i*_, ⋯, *x*_*di*_)^*t*^ for *k* = 1, ⋯, *d*. For simplicity, we use the spatiotemporal weights matrix $\bar{W}$ constructed from *q*-nearest neighbors based on the following modified spatiotemporal distance

$$\begin{array}{c} \left\{ \begin{array}{cc} d_{ij}^{ST}=\sqrt{{\left(d_{ij}^{S}\right)}^{2}+{\left(d_{ij}^{T}\right)}^{2}}, & t_{j}<t_{i}\\ d_{ij}^{ST}=\infty , & t_{j}>t_{i} \end{array}.\right. \end{array}$$ (26)

When the observation *j* is one of *q* nearest neighbors of the observation *i*, ${\bar{W}}_{ij}=1/q$, otherwise ${\bar{W}}_{ij}=0$. The diagonal elements are ${\bar{W}}_{ii}=0$. The spatiotemporal weights matrix $\bar{W}$ is row-normalized.

To develop KBGTWAR model, we apply the basic principle of the linear SVM regression to the GTWAR model (12) after preprocessing the coordinate vectors ***u***_*i*_ by a nonlinear feature mapping function *ϕ* into some feature space F. Thus, we first assume that *ρ*(***u**_i_*) and *β*_*k*_(***u**_i_*) for *k* = 0, 1, ⋯, *d* are nonlinearly related to the coordinate vector ***u**_i_* such that *ρ*(***u**_i_*) = ***w***^*t*^ *ϕ* (***u**_i_*) + *b* ∈ [0, 1] or [−1, 1], $\beta_{k}\left(u_{i}\right)=w_{k}^{t}\phi \left(u_{i}\right)+b_{k}$, where **w** and ***w***_*k*_ are the weight vectors of dimension *d*_*h*_ corresponding to *ϕ*(***u***_*i*_). Here, *ϕ* is defined in an implicit way. An inner product in feature space has an equivalent kernel such that *K*(***u***_*i*_, ***u***_*j*_) = *ϕ* (***u***_*i*_)^*t*^ *ϕ* (***u***_*j*_), provided certain conditions hold [24]. Several choices of the kernel function are possible. As mentioned before, Gaussian kernel is most widely used. Thus, we focus on the choice of an Gaussian kernel (24) in the sequel.

Using the basic idea of the linear SVM regression, we define the convex optimization problem:

$$\begin{array}{ccc} & & \min_{w,w_{k},b,b_{k},\xi ,\xi^{*}}J=\frac{1}{2}{∥w∥}^{2}+\frac{1}{2}\sum_{k=0}^{d}{∥w_{k}∥}^{2}+C\sum_{i=1}^{n}(\xi_{i}+\xi_{i}^{*})\\ & & \text{subject}\;\text{to}\;\;\{\begin{array}{c} y_{i}-(w^{t}\phi (u_{i})+b){\bar{W}}_{i}y-\sum_{k=0}^{d}x_{ki}(w_{k}^{t}\phi \left(u_{i}\right)+b_{k})\leq \;\xi_{i}\\ (w^{t}\phi (u_{i})+b){\bar{W}}_{i}y+\sum_{k=0}^{d}x_{ki}(w_{k}^{t}\phi \left(u_{i}\right)+b_{k})-y_{i}\leq \xi_{i}^{*}\\ \xi_{i},\xi_{i}^{*}\geq 0\\ 0\leq w^{t}\phi \left(u_{i}\right)+b\leq 1\;\text{if}\;\rho \in \left[0,1\right]\\ \text{or}\;-1\leq w^{t}\phi \left(u_{i}\right)+b\leq 1\;\text{if}\;\rho \in \left[-1,1\right] \end{array}, \end{array}$$ (27)

where the constant *C* > 0 is the regularization parameter and we use the notation ${\bar{W}}_{i}$ to refer to the *i*th row of matrix $\bar{W}$. For simplicity we set up the size *ϵ* of the insensitive zone to zero.

Now we are going to construct the primal Lagrange function for the case where *ρ* ∈ [0, 1] or *ρ* ∈ [−1, 1]. For the case of *ρ* ∈ [0, 1], the Lagrange function is constructed as follows:

$$\begin{array}{ccc} L=J & - & \sum_{i=1}^{n}\alpha_{i}(\xi_{i}-y_{i}+(w^{t}\phi \left(u_{i}\right)+b){\bar{W}}_{i}y+\sum_{k=0}^{d}x_{ki}(w_{k}^{t}\phi \left(u_{i}\right)+b_{k}))\\ & - & \sum_{i=1}^{n}\alpha_{i}^{*}(\xi_{i}^{*}+y_{i}-(w^{t}\phi \left(u_{i}\right)+b){\bar{W}}_{i}y-\sum_{k=0}^{d}x_{ki}(w_{k}^{t}\phi \left(u_{i}\right)+b_{k}))\\ & - & \sum_{i=1}^{n}\eta_{i}\xi_{i}-\sum_{i=1}^{n}\eta_{i}^{*}\xi_{i}^{*}-\sum_{i=1}^{n}\nu_{i}(w^{t}\phi \left(u_{i}\right)+b)-\sum_{i=1}^{n}\nu_{i}^{*}(1-w^{t}\phi \left(u_{i}\right)-b), \end{array}$$ (28)

where $\alpha_{i},\alpha_{i}^{*},\eta_{i},\eta_{i}^{*},\nu_{i},\nu_{i}^{*}$ are the Lagrange multipliers.

For the case of *ρ* ∈ [−1, 1], the Lagrange function is constructed as follows:

$$\begin{array}{ccc} L=J & - & \sum_{i=1}^{n}\alpha_{i}(\xi_{i}-y_{i}+(w^{t}\phi \left(u_{i}\right)+b){\bar{W}}_{i}y+\sum_{k=0}^{d}x_{ki}(w_{k}^{t}\phi \left(u_{i}\right)+b_{k}))\\ & - & \sum_{i=1}^{n}\alpha_{i}^{*}(\xi_{i}^{*}+y_{i}-(w^{t}\phi \left(u_{i}\right)+b){\bar{W}}_{i}y-\sum_{k=0}^{d}x_{ki}(w_{k}^{t}\phi \left(u_{i}\right)+b_{k}))\\ & - & \sum_{i=1}^{n}\eta_{i}\xi_{i}-\sum_{i=1}^{n}\eta_{i}^{*}\xi_{i}^{*}-\sum_{i=1}^{n}\nu_{i}(1+w^{t}\phi \left(u_{i}\right)+b)-\sum_{i=1}^{n}\nu_{i}^{*}(1-w^{t}\phi \left(u_{i}\right)-b), \end{array}$$ (29)

where $\alpha_{i},\alpha_{i}^{*},\eta_{i},\eta_{i}^{*},\nu_{i},\nu_{i}^{*}$ are the Lagrange multipliers.

It is understood that the dual variables in (28) and (29) have to satisfy positivity constraints, i.e., $\alpha_{i},\alpha_{i}^{*},\eta_{i},\eta_{i}^{*},\nu_{i},\nu_{i}^{*}\geq 0$. It follows from the saddle point condition that the partial derivatives of L with respect to the primal variables $\left(w,w_{k},b,b_{k},\xi_{i},\xi_{i}^{*}\right)$ have to vanish for optimality.

$$\begin{array}{ccc} \frac{\partial L}{\partial w}=0 & \to & w=\sum_{i=1}^{n}(\left(\alpha_{i}-\alpha_{i}^{*}\right){\bar{W}}_{i}y+\nu_{i}-\nu_{i}^{*})\phi \left(u_{i}\right) \end{array}$$ (30)

$$\begin{array}{ccc} \frac{\partial L}{\partial w_{k}}=0 & \to & w_{k}=\sum_{i=1}^{n}\left(\alpha_{i}-\alpha_{i}^{*}\right)x_{ki}\phi \left(u_{i}\right),\;k=0,1,\cdots ,d \end{array}$$ (31)

$$\begin{array}{ccc} \frac{\partial L}{\partial b}=0 & \to & \sum_{i=1}^{n}(\left(\alpha_{i}-\alpha_{i}^{*}\right){\bar{W}}_{i}y+\left(\nu_{i}-\nu_{i}^{*}\right))=0 \end{array}$$ (32)

$$\begin{array}{ccc} \frac{\partial L}{\partial b_{k}}=0 & \to & \sum_{i=1}^{n}\left(\alpha_{i}-\alpha_{i}^{*}\right)x_{ki}=0,\;k=1,\cdots ,d \end{array}$$ (33)

$$\begin{array}{ccc} \frac{\partial L}{\partial \xi_{i}}=0 & \to & C-\alpha_{i}-\eta_{i}=0,i=1,\cdots ,n \end{array}$$ (34)

$$\begin{array}{ccc} \frac{\partial L}{\partial \xi_{i}^{*}}=0 & \to & C-\alpha_{i}^{*}-\eta_{i}^{*}=0,i=1,\cdots ,n \end{array}$$ (35)

Classical Lagrangian duality enables the primal problem to be transformed to their dual problem. Substituting (30), (31), (32), (33), (34) and (35) into (28) and (29) yields two dual optimization problems, respectively. For the case of *ρ* ∈ [0, 1], the dual optimization problem is obtained as follows:

$$\begin{array}{ccc} & & \min_{\alpha_{i}^{\left(*\right)},\nu_{i}^{\left(*\right)}}\frac{1}{2}\sum_{i,j}({\bar{W}}_{i}y\left(\alpha_{i}-\alpha_{i}^{*}\right)+\nu_{i}-\nu_{i}^{*})K_{ij}({\bar{W}}_{j}y\left(\alpha_{j}-\alpha_{j}^{*}\right)+\nu_{j}-\nu_{j}^{*})\\ & & +\frac{1}{2}\sum_{i,j}\left(\alpha_{i}-\alpha_{i}^{*}\right)x_{ki}K_{ij}x_{kj}\left(\alpha_{j}-\alpha_{j}^{*}\right)-\sum_{i=1}^{n}y_{i}\left(\alpha_{i}-\alpha_{i}^{*}\right)+\sum_{i=1}^{n}\nu_{i}^{*}\\ & & \text{subject}\;\text{to}\;\;\{\begin{array}{c} 0<\alpha_{i}<C,\;0<\alpha_{i}^{*}<C\\ \nu_{i}\geq 0,\;\nu_{i}^{*}\geq 0 \end{array} \end{array}$$ (36)

For the case of *ρ* ∈ [−1, 1], the dual optimization problem is obtained as follows:

$$\begin{array}{ccc} & & \min_{\alpha_{i}^{\left(*\right)},\nu_{i}^{\left(*\right)}}\frac{1}{2}\sum_{i,j}({\bar{W}}_{i}y\left(\alpha_{i}-\alpha_{i}^{*}\right)+\nu_{i}-\nu_{i}^{*})K_{ij}({\bar{W}}_{j}y\left(\alpha_{j}-\alpha_{j}^{*}\right)+\nu_{j}-\nu_{j}^{*})\\ & & +\frac{1}{2}\sum_{i,j}\left(\alpha_{i}-\alpha_{i}^{*}\right)x_{ki}K_{ij}x_{kj}\left(\alpha_{j}-\alpha_{j}^{*}\right)-\sum_{i=1}^{n}y_{i}\left(\alpha_{i}-\alpha_{i}^{*}\right)+\sum_{i=1}^{n}\left(\nu_{i}+\nu_{i}^{*}\right)\\ & & \text{subject}\;\text{to}\;\;\{\begin{array}{c} 0<\alpha_{i}<C,\;0<\alpha_{i}^{*}<C\\ \nu_{i}\geq 0,\;\nu_{i}^{*}\geq 0 \end{array} \end{array}$$ (37)

Solving the above quadratic programming (QP) problem (36) or (37) with the constraints determines the optimal Lagrange multipliers ${\hat{\alpha }}_{i},{\hat{\alpha }}_{i}^{*}$ and ${\hat{\nu }}_{i},{\hat{\nu }}_{i}^{*}$. Thus, the estimated weight vectors $\hat{w}$ and ${\hat{w}}_{k}$ are obtained, respectively as follows:

$$\begin{array}{c} \hat{w}=\sum_{i=1}^{n}(\left({\hat{\alpha }}_{i}-{\hat{\alpha }}_{i}^{*}\right){\bar{W}}_{i}y+{\hat{\nu }}_{i}-{\hat{\nu }}_{i}^{*})\phi \left(u_{i}\right) \end{array}$$ (38)

$$\begin{array}{c} {\hat{w}}_{k}=\sum_{i=1}^{n}\left({\hat{\alpha }}_{i}-{\hat{\alpha }}_{i}^{*}\right)x_{ki}\phi \left(u_{i}\right),\;k=0,1,\cdots ,d \end{array}$$ (39)

Thus, for a point ***u***_*i*_ associated with the training data set D, *ρ*(***u***_*i*_) and *β*_*k*_(***u***_*i*_) are obtained, respectively as follows:

$$\begin{array}{c} \hat{\rho }\left(u_{i}\right)=(Diag\left\{\bar{W}y\right\}\left(\hat{\alpha }-{\hat{\alpha }}^{*}\right)+\hat{\nu }-{\hat{\nu }}^{*})K_{i}+\hat{b} \end{array}$$ (40)

$$\begin{array}{c} {\hat{\beta }}_{k}\left(u_{i}\right)={\left(K_{i}^{t}\circ X^{k}\right)}^{t}\left(\hat{\alpha }-{\hat{\alpha }}^{*}\right)+{\hat{b}}_{k},\;k=0,1,\cdots ,d, \end{array}$$ (41)

where ***K***_*i*_ is the *i*th row of the kernel matrix ***K*** whose elements are *ϕ*(***u***_*i*_)^*t*^ *ϕ*(***u***_*j*_) = *K*(***u***_*i*_, ***u***_*j*_), ***X***^*k*^ is the *k*th column of the *n* × (*d* + 1) design matrix ***X***, and ∘ is the Hadamard product.

Here, $\hat{b}$ and ${\hat{b}}_{k}$ can be determined by the linear regression with the input vector ${\left({\bar{W}}_{i}y,X_{i}\right)}^{t}$ and the output variable

$$\begin{array}{c} y_{i}-(Diag\left\{\bar{W}y\right\}\left(\hat{\alpha }-{\hat{\alpha }}^{*}\right)+\hat{\nu }-{\hat{\nu }}^{*})K_{i}-\left(\left(X_{i}X^{t}\right)\circ K_{i}\right)\left(\hat{\alpha }-{\hat{\alpha }}^{*}\right), \end{array}$$ (42)

which is,

$$\begin{array}{ccc} & & y_{i}-(Diag\left\{\bar{W}y\right\}\left(\hat{\alpha }-{\hat{\alpha }}^{*}\right)+\hat{\nu }-{\hat{\nu }}^{*})K_{i}-\left(\left(X_{i}X^{t}\right)\circ K_{i}\right)\left(\hat{\alpha }-{\hat{\alpha }}^{*}\right)\\ & = & \left(b,b_{0},b_{1},\cdots ,b_{k}\right){\left({\bar{W}}_{i}y,X_{i}^{t}\right)}^{t}\;\;\text{for}\;i\in I_{\text{sv}}, \end{array}$$ (43)

where $I_{\text{sv}}=\{i=1,2,\cdots ,n:0<|\alpha_{i}-\alpha_{i}^{*}\left|<C\;\;\text{or}\;\;\right|\nu_{i}-\nu_{i}^{*}\left|>0\right\}$ is obtained by exploiting Karush-Kuhn-Tucker conditions [23]. That is, the estimated values of $\hat{y}$ is obtained as follows:

$$\begin{array}{c} \hat{y}=Diag\left\{\bar{W}y\right\}\hat{\rho }+\sum_{k=0}^{d}x_{k}\circ {\hat{\beta }}_{k}, \end{array}$$ (44)

where $\hat{\rho }={\left(\hat{\rho }\left(u_{1}\right),\cdots ,\hat{\rho }\left(u_{n}\right)\right)}^{t}$ and ${\hat{\beta }}_{k}={\left({\hat{\beta }}_{k}\left(u_{1}\right),\cdots ,{\hat{\beta }}_{k}\left(u_{n}\right)\right)}^{t}$.

### Model selection

The functional structure of the KBGTWAR model is characterized by the regularization parameter *C*, the kernel parameter *κ* and the number *q* of the nearest neighbors. These hyperparameters will affect the final model complexity. We now illustrate the model selection method which determines the optimal values of these hyperparameters of the KBGTWAR model. To choose these hyperparameters we utilize the AIC function which is defined according to [25] as follows:

$$\begin{array}{c} AIC\left(\lambda \right)=\frac{RSS(\lambda )}{n}+\frac{2d}{n}{\hat{\sigma }}^{2}\left(\lambda \right), \end{array}$$ (45)

where **λ** is the vector of hyperparameters, $RSS\left(\lambda \right)=\sum_{i=1}^{n}{\left(y_{i}-{\hat{y}}_{i}\right)}^{2}$, ${\hat{\sigma }}^{2}\left(\lambda \right)$ denotes the estimate of noise variance defined as ${\hat{\sigma }}^{2}\left(\lambda \right)=\frac{1}{n-d}RSS\left(\lambda \right)$, and *d* is the number of free parameters, i.e., the size of $I_{\text{sv}}=\{i=1,2,\cdots ,n:0<|\alpha_{i}-\alpha_{i}^{*}\left|<C\;\;\text{or}\;\;\right|\nu_{i}-\nu_{i}^{*}\left|>0\right\}$.

## Case study

This section illustrates the performance of the proposed KBGTWAR model using house price data collected from 2001 to 2008 in Shenzhen, China. This data set was firstly used in [18]. Shenzhen is a special economic zone, which is situated in Guangdong Province immediately north of Hong Kong Special Administrative Region. This city forms part of the Pearl River Delta megalopolis. There are six administrative districts in Shenzhen, which are Luohu, Nanshan, Futian, Yantian, Bao’an and Longgang. By the way, the last two districts are not situated in the Special Zone. See for further details [18]. The house prices of Shenzhen continue to increase at an alarming rate due to rapid industrialization and urbanization. The study data on house prices were provided by the Shenzhen municipal bureau of land resources and housing management. From the study area 406 observations are available.

[18] reported that for empirical study thirteen input variables were used, but only six of them were statistically significant at the 90% confidence level according to their *t*-probabilities. The six input variables used in this study are land area (LANDA), distance from the nearest major road (DROAD), quality (QUAL), land price (LPRICE), property management spend (MAGT), and proximity to bus (TRAFF). The input variables and (*u*, *v*, *t*) coordinate variables are standardized such as (*z* − min(*z*))/(max(*z*) − min(*z*)). As in [26], a recent selling price is used as output variable, which stands as a proxy for the market value of the house. In fact, the logarithm of recent selling price is considered as output variable.

According to [18], there exist spatiotemporal autocorrelations in the output variable. Therefore, it is appropriate to use GTWAR-based models for this house price data set. For comparison, the global OLS model, the spatial autoregressive (SAR) model and three different GWR-based models including GWR, GTWAR and KBGTWAR are implemented using the same data set. The OLS and SAR models are employed to analyze the house price data by considering space coordinates and time as exogenous variables. GTWAR and KBGTWAR models are used to analyze the house price data with spatial and temporal considerations.

**Fig 1:**
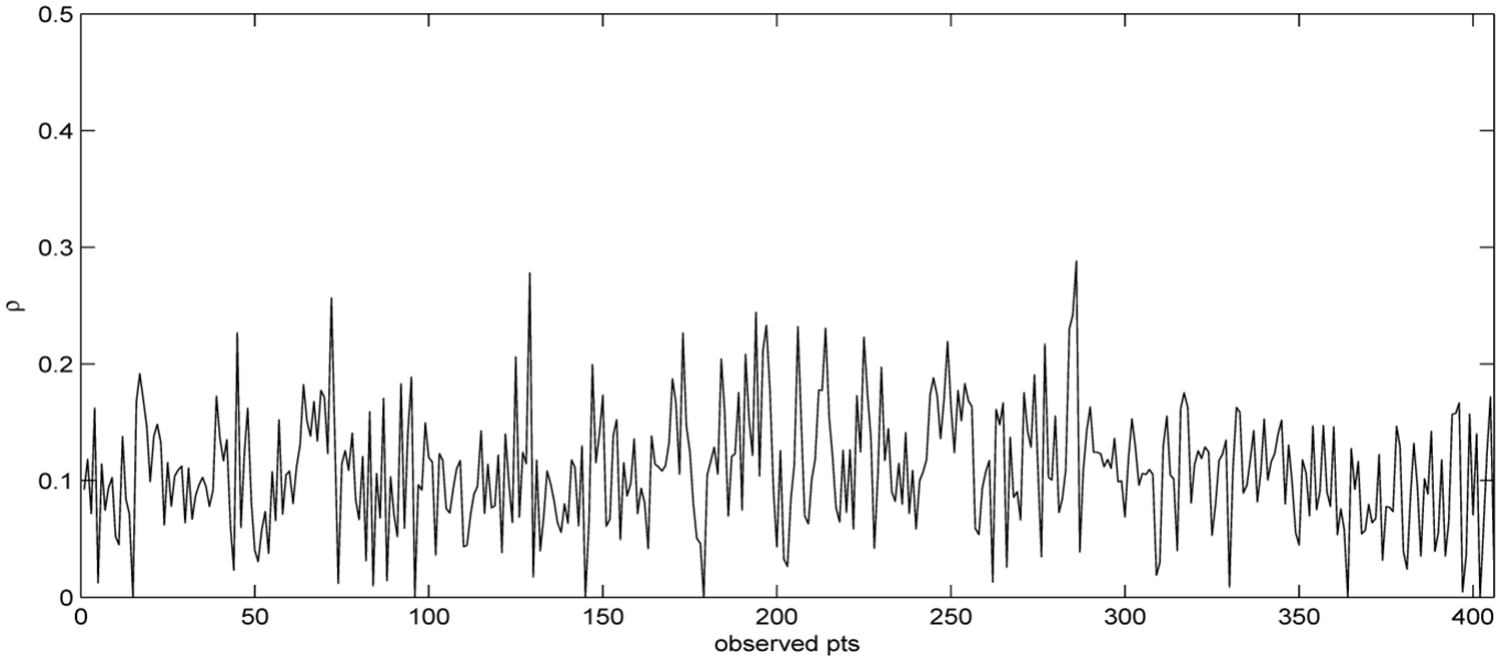
Estimated spatial autoregressive parameter. The estimated spatial autoregressive parameter(*ρ* ∈ [0, 1]) values for 406 observed points.

Using the cross validation (CV) technique, [18] determined that the optimal number of the nearest neighbors for GWR and GTWAR models is *q* = 51 and *q* = 44, respectively. In this paper we obtain *q* = 53 using the AIC technique for KBGTWAR model. As mentioned before, the spatiotemporal weights matrix $\bar{W}$ for KBGTWAR model is constructed using the spatiotemporal distance (26) instead of the spatiotemporal distance in [18]. Using this $\bar{W}$, we obtain the value of Moran’s *I* for KBGTWAR model, which is 0.1105. This value indicates that spatiotemporal autocorrelation is positive and thus we need to use KBGTWAR model under the condition *ρ* ∈ [0, 1]. We examine parameter estimates for models under consideration and goodness of fit in terms of RSS and *R*^2^. For the case of *ρ* ∈ [0, 1], the values of hyperparameters of KBGTWAR model are determined by the AIC method as (*C*, *κ*, *q*) = (50, 0.05, 53). The results are reported in Tables 1 and 2. In fact, the estimate of *ρ* for GTWAR and KBGTWAR is the median of 406 estimated *ρ*_*i*_’s. Both median values are very similar when *ρ* ∈ [0, 1]. Fig 1 reports the estimated *ρ* ∈ [0, 1] values for 406 observed points. By comparing RSS and *R*^2^ values, KBGTWAR gives significantly better fit of data than OLS, SAR, GWR and GTWAR models. The proposed KBGTWAR model provides the bigger F-statistic value than GWR and GTWAR models. Therefore, this indicates that it is more appropriate to model this particular data set with nonlinear local KBGTWAR model.

**Table 1:** Parameter estimate results of OLS, SAR and GWR for residential estate price data of Shenzhen.

	OLS	SAR (*q* = 44, *ρ* ∈ [−1, 1])	GWR (*q* = 51)		
Parameter			Min	Med	Max
Intercept	8.0027	8.426	3.519	7.713	9.663
LANDA	0.4178	0.416	-4.750	0.236	5.011
DROAD	0.3627	0.361	-2.400	-0.310	2.928
QUAL	0.2469	0.246	-15.535	-0.304	19.686
LPRICE	1.5161	1.538	-3.734	2.125	12.279
MAGT	1.5734	1.574	-3.021	2.643	6.117
TRAFF	1.0397	1.042	-3.292	0.954	4.842
*ρ*		-0.0470			
RSS	54.7	54.7		37.2	
*R*^2^	0.612	0.612		0.736	
F-statistic	69.38	69.38		1.63	

**Table 2:** Parameter estimate results of GTWAR and KBGTWAR for residential estate price data of Shenzhen.

	GTWAR (*q* = 44, *ρ* ∈ [0, 1])			KBGTWAR (*q* = 53, *ρ* ∈ [0, 1])		
Parameter	Min	Med	Max	Min	Med	Max
Intercept	0.805	8.020	16.663	6.656	7.285	7.852
LANDA	-6.699	0.571	6.192	-0.470	1.159	5.773
DROAD	-7.237	-0.016	8.884	-4.052	0.102	3.123
QUAL	-10.280	-0.941	5.490	-4.987	0.126	2.305
LPRICE	-6.377	1.300	10.584	-2.123	1.476	7.198
MAGT	-9.794	1.776	8.753	-2.363	1.702	5.819
TRAFF	-5.589	1.102	9.991	-1.792	0.656	3.109
*ρ*		0.1101			0.1088	
RSS		12.0			4.5	
*R*^2^		0.914			0.968	
F-statistic		3.84			4.45	

**Fig 2:**
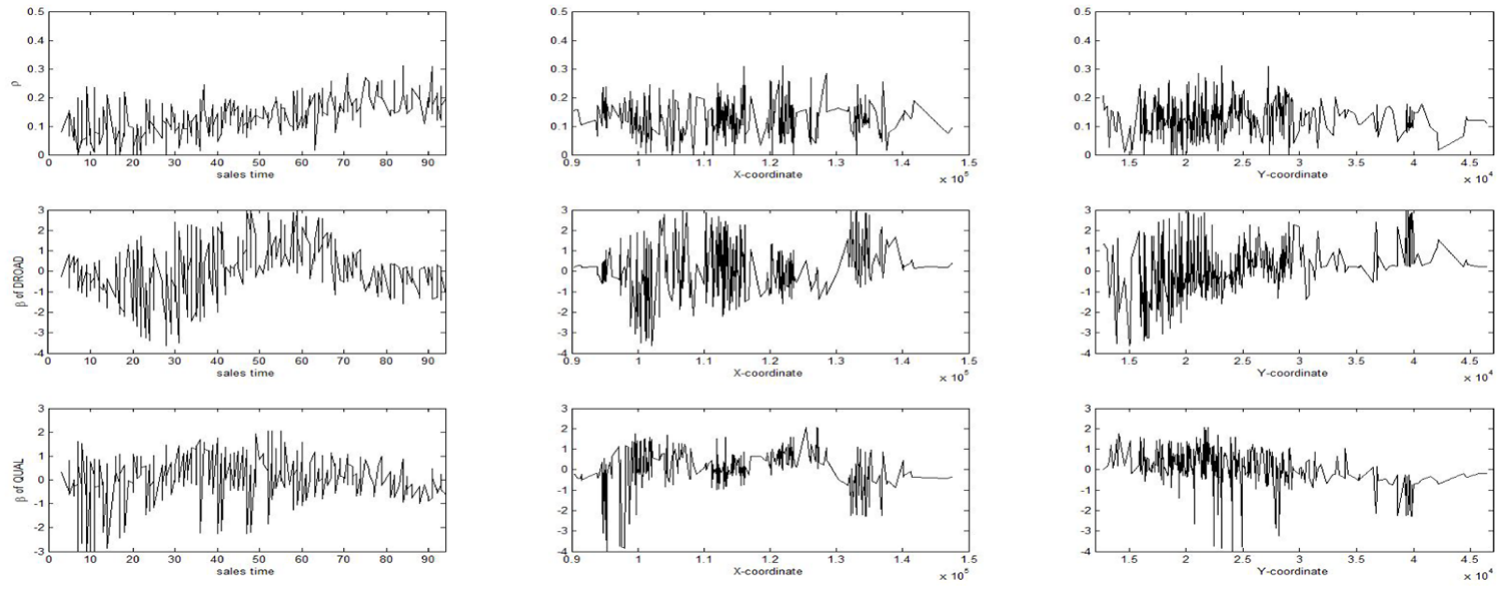
Effects of sales time and the geographical location on each individual parameter. Autoregressive parameter *ρ* (top), DROAD (middle) and QUAL (bottom) coefficients on the sales time and the *X*, *Y* coordinates of the geographical location.

**Fig 3:**
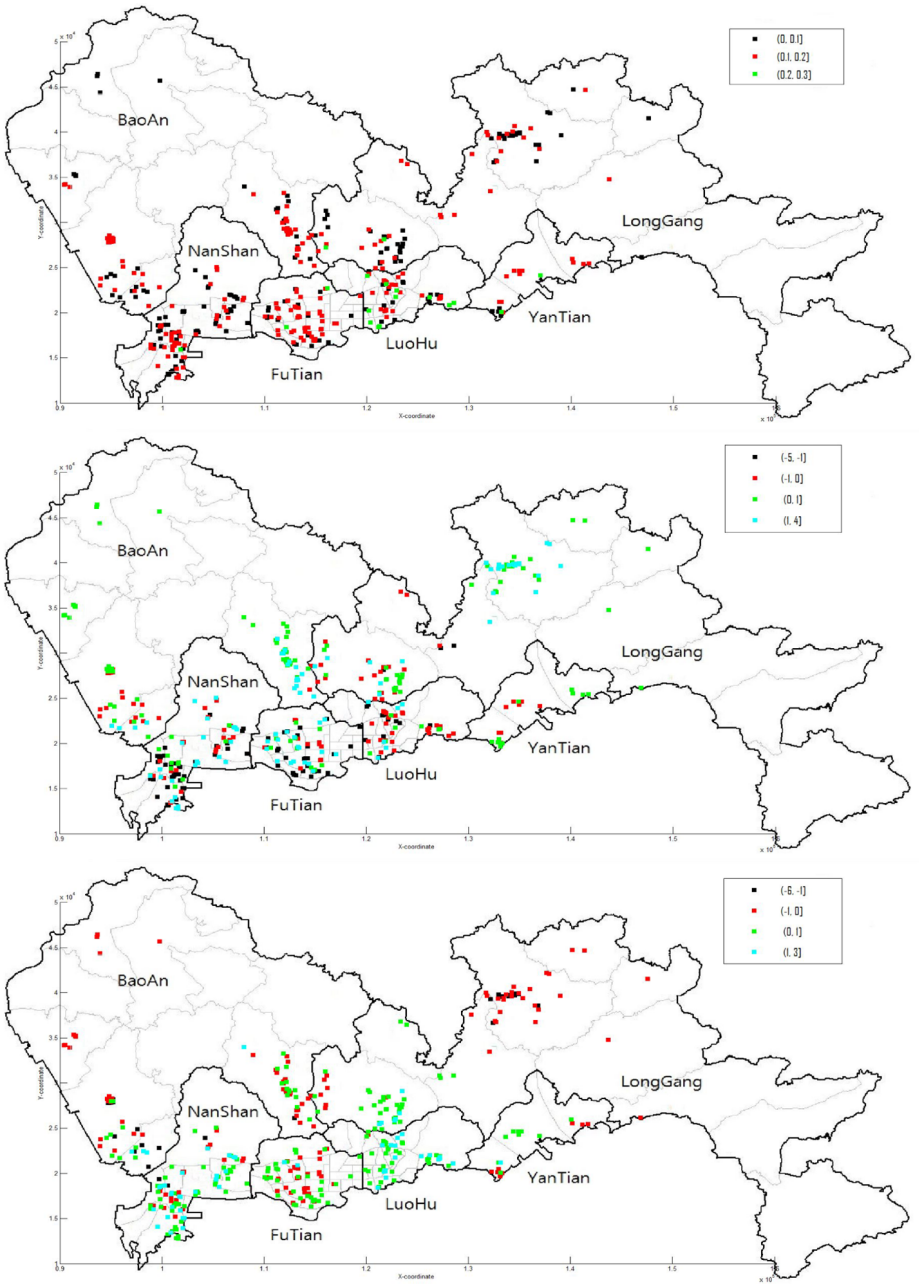
Spatial variation of each individual parameters. Autoregressive parameter (top), DROAD (middle) and QUAL (bottom) coefficients.

We now investigate the spatial and temporal variations of three selected parameters, i.e., autoregressive parameter *ρ*, DROAD and QUAL coefficients. Figs 2 and 3 show the results. The plots in Fig 2 show the effects of sales time and the geographical location on each individual parameter. As seen from Fig 2, on the whole the autoregressive parameter and QUAL coefficient values do not show the apparent spatial and temporal variations. However, DROAD coefficient show somewhat apparent spatial and temporal variations. In particular, this coefficient changes nonlinearly in the sales time and *Y*-coordinate. The maps in Fig 3 show the distribution of each individual coefficient. As seen from Fig 3, the autoregressive parameter and QUAL coefficient do not show apparent spatial variations. However, DROAD coefficient changes from low in the south to high in the north. From Figs 2 and 3 we observe that the effect of DROAD coefficient on the house price depends on the location and the sales time.

## Conclusions

In this paper, we proposed the KBGTWAR model to simultaneously account for spatiotemporal nonstationarity and autocorrelation that exist in the house prices. To devise KBGTWAR model we applied kernel technique to spatially and temporally varying coefficients and then utilized the basic principle of linear SVM to estimate the relevant parameters. Unlike GTWAR model, KBGTWAR model is basically nonlinear. Therefore, KBGTWAR model can deal with complex nonlinear trends, which are very common in spatiotemporal phenomena. KBGTWAR model can also appropriately explore the dynamic relationship between the output variable and the input variables, since this model is based on varying coefficients model which efficiently describes dynamic patterns of a regression relationship.

KBGTWAR model takes over all advantages of SVM and varying coefficients model that capture nonlinearities in the data, that have good prediction ability, and that are useful tools when the functional form of the relationship between the output variable and the input variables is left unspecified. In particular, KBGTWAR model works well under settings without strong assumptions on the distribution of the data. KBGTWAR model is also interpretable, since varying coefficients model is meaningfully interpretable.

However, as with all SVM-related models, KBGTWAR model requires lots of computing time in determining the optimal hyperparameters, and has serious computational problem for large data because it has to solve a large-scale quadratic programming problem to get the values of relevant parameters. These are disadvantages associated with KBGTWAR model.

This paper analyzed data reflecting the spatiotemporal nonstationarity and autocorrelation of house prices. OLS, SAR, GWR, GTWAR and KBGTWAR models used in the study were built based on house price data collected between 2001 and 2008 in the city of Shenzhen, China. The performances of these models were then compared based on RSS, *R*^2^ and F-statistic. This paper demonstrates that the proposed KBGTWAR model provides good results in goodness of fit for the given example. To conclude, we proposed a more efficient KBGTWAR model to account for spatiotemporal nonstationarity and autocorrelation simultaneously.
